# Birds differentially prioritize visual and olfactory foraging cues depending on habitat of origin and sex

**DOI:** 10.1098/rsos.221336

**Published:** 2023-02-08

**Authors:** Diana Rubene, Matthew Low, Anders Brodin

**Affiliations:** ^1^ Department of Crop Production Ecology, Swedish University of Agricultural Sciences, Uppsala, Sweden; ^2^ Department of Ecology, Swedish University of Agricultural Sciences, Uppsala, Sweden; ^3^ Department of Biology, Lund University, Lund, Sweden

**Keywords:** great tit, urbanization, learning, bird olfaction, multisensory integration

## Abstract

Animals interpret their environment by combining information from multiple senses. The relative usefulness of different senses may vary between species, habitats and sexes; yet, how multimodal stimuli are integrated and prioritized is unknown for most taxa. We experimentally assessed foraging preferences of great tits (*Parus major*) to test whether urban and forest individuals prioritize visual and olfactory cues differently during foraging. We trained 13 wild-caught birds to associate multimodal (colour + odour) cues with a food reward and assessed their foraging preferences in a cue-separation test. In this, the birds could choose between the multimodal training cue and its olfactory or visual components. Our results suggest that the birds did not perceive multimodal cues in an integrated way, as their response was not stronger than for unimodal cue components. Urban birds preferred olfactory cues, while forest birds preferred visual cues. Nevertheless, female birds preferred the multimodal cue, while males foraged more randomly with respect to which cue was present. These findings contribute to our understanding of the relative roles of vision and olfaction in bird foraging behaviour. Future work should focus on how habitat- and sex-specific sensory prioritization modifies bird foraging behaviour and foraging success in the context of urban adaptations across populations.

## 1. Introduction

In a complex and changing environment, animals rely on the information provided by their senses for finding food, avoiding predators and locating potential mates [1]. When using environmental information for decision-making, animals must often combine inputs from more than one sensory system (e.g. visual, auditory and olfactory cues). Multisensory integration describes the cognitive processes whereby multimodal information interacts to form an animal's perceptual experience [2]. During these processes, different modalities can facilitate each other, resulting in improved signal detection and response [3], or one modality may be prioritized over another [2,4]. How multimodal information is integrated and used in animals’ daily lives is still poorly known for most taxa, but such knowledge could contribute to novel insights about individual behaviour and environmental adaptations.

When encountering multimodal stimuli, animals may focus their attention on and prioritize different senses under specific conditions [3,5,6]. Sensory prioritization can be influenced by bottom-up factors, which are automatic and signal driven, such as salience or low detection threshold, or top-down factors, which are voluntary and linked to previous experience, goals and expectations of the receiver [2]. Also, the reliability of specific senses may be context dependent, e.g. if processing is more efficient in one modality than the other for a specific task, known as the ‘modality appropriateness’ principle [7]. Modality appropriateness may shift if the signal in one modality is masked or disturbed under changing environmental conditions or presence of environmental noise [4,8]. Evaluating how animals prioritize inputs from their senses during ecologically relevant tasks could be important for understanding how they adapt to environmental changes, e.g. urbanization. However, even in extensively studied taxa like bees, there is still a lack of evidence on how environmental sensory noise affects use and usefulness of multimodal information [4,8,9]. In other taxa like birds, we are only now at a stage of starting to pose questions and formulate hypotheses in this field [10].

An interesting context for studying sensory prioritization is urbanization. Selecting relevant information is an important aspect of animals' adaptation to the urban habitat, where disturbing stimuli, like light, noise and volatile chemicals, act as interfering sensory pollutants [11,12]. Urban populations often differ from those in natural habitats in behaviour, personalities and life histories (reviewed in [13]). The behaviour of urban birds is influenced by anthropogenic noise and light, with negative fitness consequences; these sensory pollutants have also been linked to bird signalling and sensory perception [11]. In addition, urban areas contain high levels of volatile chemicals [14] that may act as olfactory sensory pollutants, but possible effects of anthropogenic odours on birds have not been studied.

Birds use odour cues in many contexts and olfaction is becoming increasingly recognized as important for the understanding of how they interact with the environment [15]. Insectivorous species such as great tits (*Parus major*) can use herbivore-induced plant volatiles (HIPVs) to identify caterpillar-infested trees when foraging [16,17]. This ability is important for their foraging success because herbivorous larvae are a major part of nestling diet [18]. Urban birds fledge smaller and fewer offspring [19,20], likely because of food limitation during breeding, as urban environments host a lower abundance of high-quality larval food [18]. In addition, volatile air pollutants can impair odour-mediated interactions between plants, herbivores and their predators [21], and they may interfere with birds’ ability to use HIPVs to locate food in urban habitats. This could potentially result in olfaction being downgraded compared to vision in urban birds. Thus, how birds prioritize senses in different habitats could have implications for their foraging success.

The role of olfaction may also differ between sexes. Olfactory ability has been linked to hormonal activity in males of a related species, the blue tit (*Cyanistes caeruleus*), suggesting potentially a higher olfactory sensitivity in males than in females during breeding [22]. In the context of foraging, however, the importance of olfaction should be similar for females and males as both parents contribute to nestling provisioning. Outside of the breeding season, competition for food may potentially alter foraging strategies, because males dominate females at food sources [23], which might influence how birds of different sex use sensory information.

In this study, we aimed to understand how birds perceive multimodal stimuli in a foraging context by estimating their relative reliance on visual versus olfactory cues. In insects, particularly bees, integration of olfactory and visual information via bottom-up processes is relatively well studied (reviewed by [8]): in summary, multimodal stimuli have higher salience than unimodal stimuli, attract attention more effectively, improve signal detection and increase discrimination accuracy [3,8,24–26]. There is currently no evidence that this type of multisensory integration occurs in birds [27], and a previous study from our group suggests that great tits do not learn multimodal forging cues faster than either visual or olfactory cues alone [28]. In that study, however, we compared learning speed between birds that were trained with only one type of stimulus (visual, olfactory or multimodal) [28]; it is still possible that because great tits are good learners they may be able to use unimodal and multimodal signals equally well if required. Yet, other aspects of multisensory integration may be uncovered by examining how birds perceive and prioritize components of multimodal cues. Therefore, in this study, we aim to assess whether birds that have been trained to associate multimodal cues with food perceive them in combination or prefer one modality to the other, which would suggest that individuals differ in sensory priorities. To set sensory prioritization in an ecologically relevant context, we also wanted to test whether birds from a city or forest environment or females or males had different sensory preferences.

We chose to study sensory foraging preferences in the great tit, because great tits in Europe have colonized urban areas, but also remain in their natural forest habitats. This makes it a suitable model species for studying urban adaptations. Great tits use both vision and olfaction during foraging [29], and will quickly learn cues associated with either sense [28]. In the present study, we asked the following questions: (1) do great tits learn and perceive components of multimodal cues in an integrated way and (2) do urban and forest great tits, or females and males, prioritize vision and olfaction differently during foraging?

We expected that bird preferences for multimodal cues should be consistently higher than for the individual components (or alternatively be the highest at the start of experimental trials), if they perceive multimodal information in an integrated way. This is due to potentially higher salience, faster processing and more efficient response to multimodal information reported for other taxa [2,5,8,25]. We also expected that urban birds might show relatively lower priority for olfaction. This is because their olfactory function may have experienced interference by urban air pollutants, according to hypotheses suggested previously [9,21]. We further expected that female and male birds may have different priorities because sexes experience different selective pressures and cognitive differences between female and male great tits have previously been reported in foraging contexts [30]. In addition, sex differences are known to occur in other cognitive and behavioural traits in the great tit and other bird species [31,32].

## 2. Methods

### 2.1. Birds

We used 13 wild-caught adult great tits: six urban (three females and three males) and seven forest birds (three females and four males). We captured the birds outside of the breeding season (November 2019–February 2020) using mist nets. We pre-established bird-feeding stations at the capture sites to increase overall bird activity. During capture, we switched the location of the bird feeder to facilitate bird movement between the old and the new location and placed the mist net between these. In addition, audio playback was used to attract individuals from the surrounding area. We used two urban localities in the city of Lund, Sweden (55°42′52″N, 13°12′26″E; 55°41′53″E, 13°14′60″N) and three forest/rural localities in the area of Höör, about 30 km from Lund (55°55′18″N, 13°27′11″E; 55°53′27″N, 13°36′57″E; 55°52′34″N, 13°35′47″E). It is unknown whether great tits in the two study areas constitute genetically distinct populations. The urban localities were located at the Lund university campus and in an urban residential area. The forest localities were in rural areas dominated by deciduous and mixed forests, and agricultural land. We selected and categorized the localities as ‘urban’ and ‘forest/rural’ based on predominant land uses within 1 km radius. The urban localities had less than five percent area with forested land use and the forest/rural localities had less than five percent area with urban land use. Air pollution levels in the city of Lund are around 18 µg m^–3^ for NO_2_ and 15 µg m^–3^ for P_10_ particles (yearly averages), while in the rural area where our sites were located, the levels of these pollutants are considerably lower (less than 5 µg m^–3^) [33].

**Figure 1.**
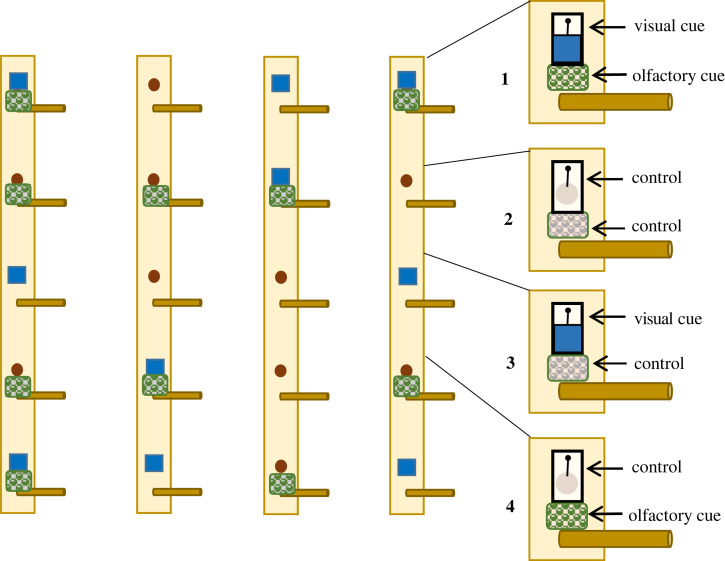
Foraging arena setup for testing sensory preferences in great tits: the arena consisted of four wooden boards with foraging holes and perches for landing. During the cue separation test, birds were presented with four options of colour and odour cues attached to the poles: 1, multimodal (colour + odour); 2, control (no cues); 3, visual (colour); and 4, olfactory (odour). White covers and odourless ‘odour bags’ were always used for control holes (as in images 1–4 on the right), but were omitted in the illustration of the arena for clarity.

We assigned each bird to a treatment group and a specific training cue before releasing them from bird bags into cages in the laboratory, without any prior knowledge about their behaviour or sensory preferences, and initially their sex. At later stages of the experiment we assigned cues to balance the number of birds of each sex or origin that were trained with specific cues; still the assignment was done before the birds were released into the experimental arena for the first time. The birds were trained and tested individually in an experimental foraging arena that contained four artificial trees with five foraging holes and perches each (figure 1). Additional details on bird housing, construction of the arena and preparation of visual and olfactory foraging cues are described in electronic supplementary material, appendix S1, and previously reported in [28].

The birds used in this study were a subset of individuals used in our previous study [28]; that study compared how fast great tits learned olfactory, visual and multimodal foraging cues. In the present study, we tested all birds that had been assigned to the ‘multimodal’ learning group in [28]. We also included one bird from the ‘visual’ group (the best-performing bird that had learned additional cues, including a multimodal cue). The cue prioritization tests were performed after the birds had completed learning tasks reported in [28]. To test cue prioritization, it was necessary that the birds had been trained with multimodal cues; therefore only these birds were included in the present study. All groups of birds were trained and tested in parallel; they came into the laboratory at the same time of season and after experiencing the same environmental conditions. This means that we can directly compare conclusions of the previous study on learning speed and olfactory preferences in the ‘visual’ and ‘olfactory’ group to the prioritization patterns observed for the ‘multimodal’ group in the present study. The experiments were terminated just before the start of the breeding season to avoid any hormonal influence on bird behaviour. This restriction limited the number of birds that we could test. We performed all experiments according to a permit from the Swedish regional ethical permit board for animal experiments (permit number 5.2.18-04716/2018).

### 2.2. Experimental procedures

To test sensory preferences, we designed a foraging task where birds had to search for food in the experimental arena using multimodal (colour + odour) foraging cues. We then assessed their relative preferences for olfactory and visual components of the multimodal cues in a cue separation test. In addition, to assess whether the birds preferred multimodal cues to unimodal ones, which would suggest an integrated perception, we also assessed changes in preference for multimodal cues over the course of experimental trials.

Prior to testing, we trained the birds in a sequence of procedures [28] (electronic supplementary material, appendix S1). First, we trained them to forage in the arena and retrieve pieces of mealworms from the foraging holes (habituation phase). Then, we trained the birds to associate multimodal foraging cues with a reward (associative learning phase). During the learning phase, we allowed birds to forage in the arena with 50% of the foraging holes baited with a cue and a reward in repeated sessions until they made five consecutive correct (cue) choices. After this, we performed a cue separation test (test phase), where equal proportions of the foraging arena contained holes baited with multimodal cue, its visual and olfactory components, and control holes. We changed the placement of the rewarded cue and control randomly for each training/testing session and for each bird. We recorded bird choices by direct observation and by video camera.

#### 2.2.1. Habituation and learning

During the habituation phase, the birds were individually trained to collect rewards that consisted of small pieces of mealworms in the foraging arena. For this, we released the birds one at a time into the arena and allowed them to explore for up to 20 min. All foraging holes contained pieces of mealworms that were fully visible during the first sessions, but later covered with small pieces of laminated paper, so that the birds had to retrieve the rewards from behind the covers. No sensory cues were used in the arena during the habituation phase. Initial olfactory preferences were assessed for each bird directly after habituation in [28] in order to control for confounding effects on cue learning, but no significant difference was found [28]. We additionally compared the initial olfactory preferences for birds of different sex and origin for the subset of birds used in the present study (see Analyses section).

During the learning phase, we trained the birds to associate food with a colour–odour combination. Each bird was assigned a single combination of one out of three colours (blue, yellow and green) and one out of three odours: methyl jasmonate (MeJA), methyl salicylate (MeSA) and vanilla, *Vanilla planifolia*. We followed a similar procedure as in the habituation phase, but now only half of the foraging holes in the arena were baited with colour–odour cues and mealworms. The other half of the holes had white control covers and no mealworm. We allowed the birds to forage in up to 20-min sessions and recorded each visit to the perches/holes until they passed a learning criterion—five consecutive correct (= perches with cues) choices in the arena (e.g. [34]). At this point, we considered that the bird had successfully formed an association between food and the multimodal cue. The birds reached the learning criterion in 2.4 learning sessions on average (range 1–9); data on habituation, initial olfactory preferences and learning for each individual are summarized in electronic supplementary material, table S1 and appendix S2.

Birds that appeared to have low motivation to search for food (e.g. did not approach the arena, sat immobile for long periods, or mostly explored other parts of the room) were food-deprived for up to two hours prior to the coming experimental sessions (30 out of 59 learning sessions). We tested the correlation between food deprivation time and bird performance to ensure that this method worked as we had intended and evened out the motivation among birds (see the Analyses section).

Assessment of learning in birds varies widely between studies, with some studies using three [35], 5–6 [34] or 7–10 consecutive correct choices [36,37]. We considered that five correct choices would be a sufficient criterion for our test setup, where the birds do not have complete information on all surrounding options at any given time. In addition, more strict criteria might exclude individuals with certain personalities, e.g. shy or fearful birds, and we wanted our sample to be as representative of the natural population as possible. However, in 19 out of 25 learning tasks, the birds performed more than five consecutive correct choices during their final session (electronic supplementary material, table S1). We additionally assessed learning progress by comparing bird performance, measured as proportion of correct choices, between the first learning session and the final session (when the bird passed the criterion).

#### 2.2.2. Cue separation test

We tested preferences for multimodal cues that the birds had learned and their unimodal components in a single cue separation test, conducted after the birds had completed the associative learning phase. Out of the 20 foraging holes, we randomly selected and baited them with cues as follows: five multimodal (visual + olfactory), five visual, five olfactory and five controls. Similar tests on insects usually split the multimodal cues and assess preferences for each modality; however, interactions between modalities may only occur when the cues are presented together [8]. Therefore, we also included the multimodal stimulus in our setup. Only the holes with multimodal cues contained food rewards to reinforce the learned stimulus and motivate birds to continue search for food. The reason was that birds at this stage lost interest quickly when they were no longer finding food. The smell of mealworms could not be used by birds as a cue, because all foraging holes were baited daily at some point and had accumulated smell over the course of the experiment; the holes were also covered, limiting the spread of the smell and making it unlikely to be detectable at a distance (before a bird landed on a perch). We released each bird into the foraging arena and allowed it to forage for up to five minutes. We recorded which perches the bird landed on and which cues these perches were associated with. We used landing on a perch as a choice, instead of probing for food in the hole, partly because several birds (presumably the shy/fearful ones) were cautious toward hole covers and did not always touch the board in attempt to probe for food. It may have also been possible for birds to visually inspect the hole from a very close distance as the hole covers hang flexibly and there could be a few millimetre gap between the board and the cover. Due to this difficulty to reliably define what behaviour constituted probing, we used landing on a perch and assumed that this action indicated the intention of the bird to search for food.

Colleagues who were not involved in experiments and were blind to the experimental treatments/design (see acknowledgements) viewed video recordings, and we compared the data from video viewing with data obtained by direct observation to confirm that they were scored correctly. We summarized the choices for each cue category (multimodal, visual, olfactory and control) and used the first 20 choices of each bird for analysis of preference, with the exception of a single bird that only performed 13 choices.

Twelve birds were trained with two different multimodal cues, i.e. different colour–odour combinations, and one bird was trained with only one multimodal cue [28] (electronic supplementary material, table S2). For seven birds, we performed cue separation tests with both the first and the second learning cue. This was done to obtain a balanced design in terms of the number of colour and odour combinations tested, as using several colour–odour combinations decreases the risk that observed preference patterns might be due to properties of a specific colour–odour combination, if individual colours and odours vary in perceived salience [38]. Thus, four birds were tested with the first learning cue, two birds with the second learning cue and seven birds with both cues. This resulted in data from 13 birds performing a total of 393 foraging choices, during 20 cue separation tests.

#### 2.2.3. Possible confounding factors

We checked that the colours and odours were as balanced as possible between groups of urban–forest and female–male individuals (electronic supplementary material, table S1 and appendix S2). However, there were still some potentially confounding factors. According to a previous experiment birds may learn MeSA odour more slowly than other odours [28] and they learn yellow colour faster than blue (D.R. 2020, unpublished data). Learning speed could potentially be related to cue salience, which means that birds trained with either MeSA or yellow might show relatively lower preference for olfactory cues and a higher preference for visual cues. To assess whether relative preferences for visual and olfactory cues differed for birds tested with yellow or MeSA, we ran additional analyses to minimize the risk that the patterns in our observations did not result from this potential bias (see Analyses section).

During the search for mealworms, some birds removed the coloured hole covers from the board. This could potentially affect a bird's perception of such a perch during a repeated visit: the bird could return because it remembered that there was a colour cue, or alternatively, it might have no memory of the cue and perceive the perch as control (or as olfactory for multimodal cues). To control for this, we rescored the data by excluding visits to foraging holes with covers removed, since what these represent to the birds was uncertain, and assessed this with additional analyses (see Analyses section).

We used 13 great tit individuals in our experiment, which might be considered a low sample size. Since training animals to perform learning tasks is time-consuming, cognition studies frequently use relatively few individuals and sample sizes less than 10 are common (e.g. [37,39]), which creates a risk that those individuals do not represent the broader natural population [40]. Therefore, we used the STRANGE framework proposed by [40] to assess our group of birds and experimental procedures and identified no obvious sources of potential bias (electronic supplementary material, table S2 and appendix S2).

### 2.3. Analyses

#### 2.3.1. Habituation and learning

All analyses were performed using R software [41]. Prior to our analyses of foraging preferences, we assessed whether aspects of our methodology or individual bird behaviour/preferences might have influenced performance in the cue separation tests. To assess whether food deprivation influenced performance we used a generalized linear mixed model with Gamma distribution and a log link in package *lme4* [42]. We included data from all learning sessions to correlate proportion of correct choices with food deprivation time as a fixed factor. We added bird identity as random factor, but it explained zero variation and the model showed singularity; therefore, the random factor was subsequently removed. To assess pre-existing olfactory preferences before learning, we used linear models with the proportion olfactory cue choices as response variable, and origin and sex as fixed factors. Finally, to assess learning of the multimodal cues, we used a linear model with proportion of correct (cue) choices as response variable and learning session (first or final) as fixed factor.

#### 2.3.2. Preferences for different foraging cues

We analysed bird preferences for the different foraging cues by first estimating the frequency with which the birds chose each type of cue (control, visual, olfactory or multimodal). Since our response variable had four categories, we estimated preferences for foraging cues using a 4-level multinomial statistical modelling framework. The models were implemented in a Bayesian framework to allow us to extract the probabilities and confidence intervals of interest for each foraging cue type depending on bird sex and origin. The models were implemented in R using JAGS [43], using minimally informative priors. All models were checked for convergence by visual inspection of stability and mixing of the chains (see electronic supplementary material, appendix S3, for details of model structures and code).

The response variable was the number of choices for each cue category made by each individual during the cue separation tests. Within model structure, we estimated general preferences of the entire group of birds (model 1). We then extended the model to generate estimates of each foraging choice as influenced by capture origin (urban versus forest) and sex (female versus male) (model 2). Effects of origin and sex were estimated separately, i.e. the birds were grouped either by sex or by origin in each analyses; thus, we did not test the interaction between these two factors because adding complexity would require more data. As we used 13 birds to perform 20 cue separation tests, we ran the analyses of sex and origin (model 2) both by using each cue separation test as an individual sample (*N* = 20) and by summing the foraging choices per each individual (*N* = 13), as six birds performed two tests with different multimodal cues.

For the multimodal cues, we also estimated differences in preference between the first 5 choices and subsequent 15 choices. We did this to assess if the multimodal cue was the initial choice of the birds and perceived as the most attractive option, which may indicate an integrated perception of visual and olfactory information. We did this by extending model 2 to incorporate the order of choices and estimate changes in bird preference for the multimodal cue over time, by dividing the total 20 choices into groups of five (1–5, 6–10, 11–15, 16–20) (model 3).

#### 2.3.3. Differences in preference

We used the estimated choice frequencies and confidence intervals (Bayesian credible intervals, BCIs) for each cue type to compare preferences between the different cue types. We estimated pairwise differences for all cue contrasts within each analysis, i.e. control versus visual, control versus olfactory, visual versus olfactory, and so forth. Bayesian framework is particularly useful here, because the difference between two groups (e.g. is preference for olfaction > visual?) can be calculated by simply subtracting one posterior distribution from another (i.e. difference = olfaction − visual) [44]. All variables derived in this way are themselves probability distributions with mean values, standard errors and confidence intervals. Thus, parameters and derived variables (in our case, the estimated differences between cue preferences) where the posterior distributions do not overlap zero to a large degree can be considered to indicate clear differences between groups [44,45]. This assessment could be seen as indicating the ‘significance’ of the difference, except that in the Bayesian framework there is no cut-off value; rather the actual probability is estimated, which can then be evaluated and judged depending on the study question. We have in this study chosen to highlight contrasts for which the certainty of a real difference between groups is ≥ 95%.

#### 2.3.4. Possible confounding factors

To control for confounding variables, we ran additional analyses to verify the robustness of our results with regards to these possible confounds: (i) model 1 with only using data from birds tested with colour yellow or odour MeSA and (ii) model 2 with foraging choices scored by excluding visits to foraging holes with coloured covers removed. The additional analyses performed to control for confounding factors yielded qualitatively similar results with regard to the observed patterns in preference between urban and forest birds, and between sexes (electronic supplementary material, tables S3–S6 and appendix S4). No differences between relative preference for visual and olfactory cues were found for birds trained with odour MeSA or colour yellow (electronic supplementary material, table S3 and appendix S4). In addition, we examined whether particularly high/low values in the data may be regarded as outliers, in order to evaluate if these may affect our interpretation of the main results. We identified potential outliers using boxplots (i.e. the interquartile range method).

## 3. Results

### 3.1. Habituation and learning assessment

Food deprivation time was uncorrelated to performance measured as the proportion of correct choices per session, suggesting that this method worked as we had intended and evened out the motivation among birds (GLM: Estimate = 0.02, s.e. = 0.04, *t* = 0.5, *p* = 0.62). Urban birds tended to have a slightly higher initial olfactory preference than forest birds (mean urban = 0.58, mean forest = 0.48), but the difference was not significant (LM for the ‘multimodal’ group: Estimate = 0.07, s.e. = 0.05, *t* = 1.396, *p* = 0.19). Birds made on average 85% correct choices during the final session (mean ± s.d.: 0.85 ± 0.12) and their performance had improved significantly compared to the first session (mean ± s.d.: 0.61 ± 0.13) (electronic supplementary material, figure S2; LM: Estimate = 0.35, s.e. = 0.063, *t* = 5.502, *p* = 2.21 × 10^−6^).

### 3.2. Sensory preferences

**Figure 2.**
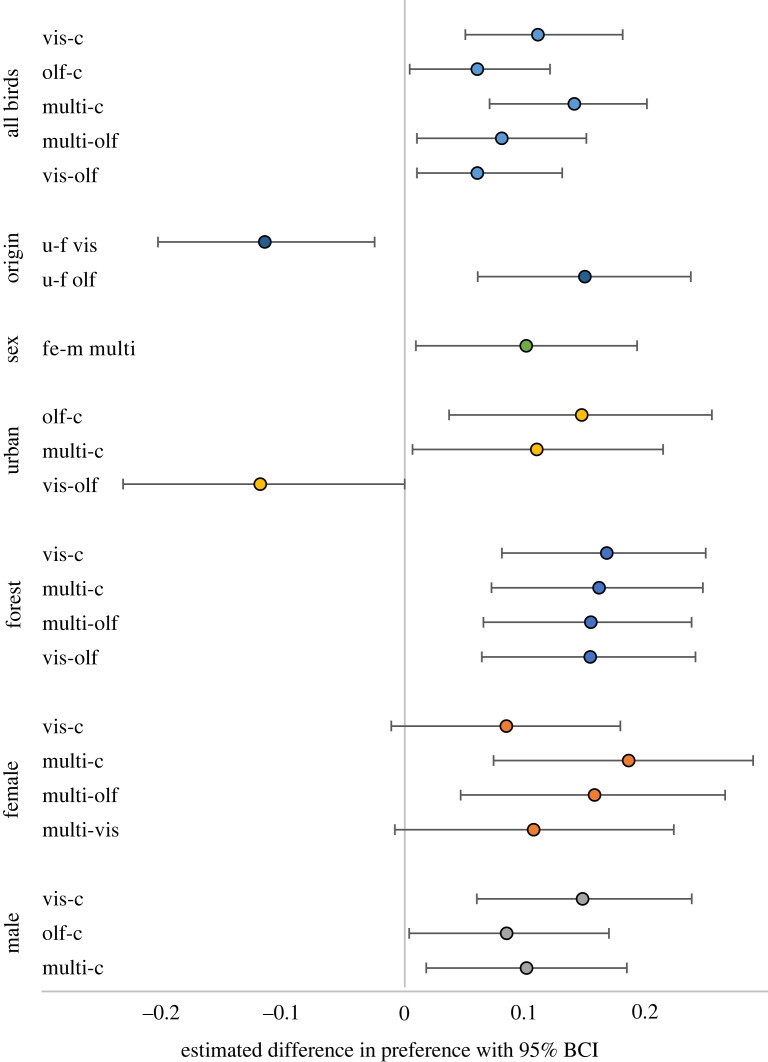
Estimated differences in great tit preference for different types of foraging cues (control = c, visual = vis, olfactory = olf, multimodal = multi), with 95% BCIs. Preferences were estimated for all birds combined (cue contrast ‘all birds’) and separately for birds of different sex (contrasts ‘sex’, ‘female’, ‘male’; female = fe, male = m) and origin (contrasts ‘origin’, ‘urban’, ‘forest’; urban = u, forest = f), and modelled using multinomial likelihood in a Bayesian framework. Only the pairwise contrasts for which the probability of a difference exceeds 95% are shown; the probabilities were calculated by estimating the proportion of the posterior distribution for the specific difference that overlaps zero.

The great tits learned and responded to both visual and olfactory foraging cue components as they showed a higher preference for perches with at least one of these cues compared to controls (figure 2 ‘all birds’). Birds had the highest preference for multimodal cues (estimated mean = 0.31, 95% BCI: 0.26–0.36) and nearly as high a preference for visual cues (estimated mean = 0.28, 95% BCI: 0.24–0.33); their overall preference for olfactory cues was lower (estimated mean = 0.23, 95% BCI: 0.19–0.28), but still clearly higher than for control (estimated mean = 0.17, 95% BCI: 0.14–0.21) (figure 2).

#### 3.2.1. Preference for multimodal cues

**Figure 3.**
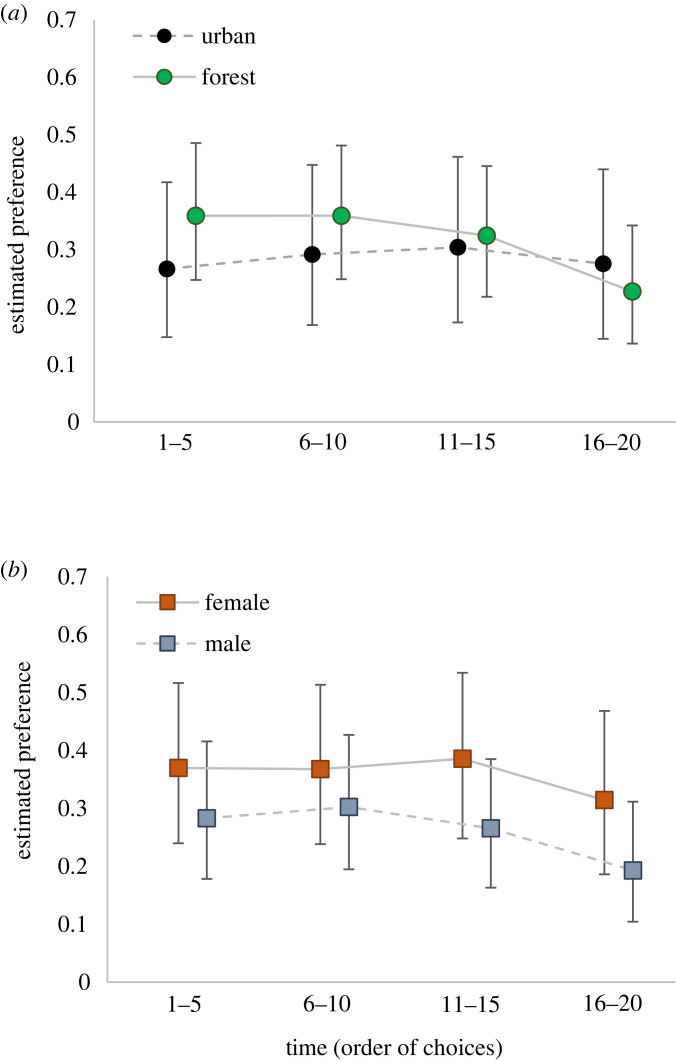
Model estimated preference (frequency of choices) of great tits for multimodal foraging cues over time, with 95% BCIs. Separate analyses were performed for urban and forest birds (*a*) and females and males (*b*). The total 20 choices that each bird performed over the course of a cue separation test were divided into four groups: 1–5, 6–10, 11–15 and 16–20. The probability of a difference did not exceed confidence level of 95% for any of the comparisons between time points.

We found that overall bird preference for the multimodal cues was not consistently higher compared to unimodal cues (figure 2 and figure 3*a*,*b*; electronic supplementary material, table S6 and appendix S4). We found no difference (estimated confidence levels < 95%) between preferences for the multimodal cue during the start of the test trials (choices 1–5) and subsequent choices (6–10, 11–15 and 16–20) (figure 3*a*,*b*). In forest birds, there was a tendency for reduced preference for multimodal cues over time, with an estimated 94% probability of a difference between the first and the last time point (figure 3*a*). A similar pattern was observed for male great tits, with an estimated 88% probability of a difference between the first and the last time point (figure 3*b*).

#### 3.2.2. Urban versus forest birds

**Figure 4.**
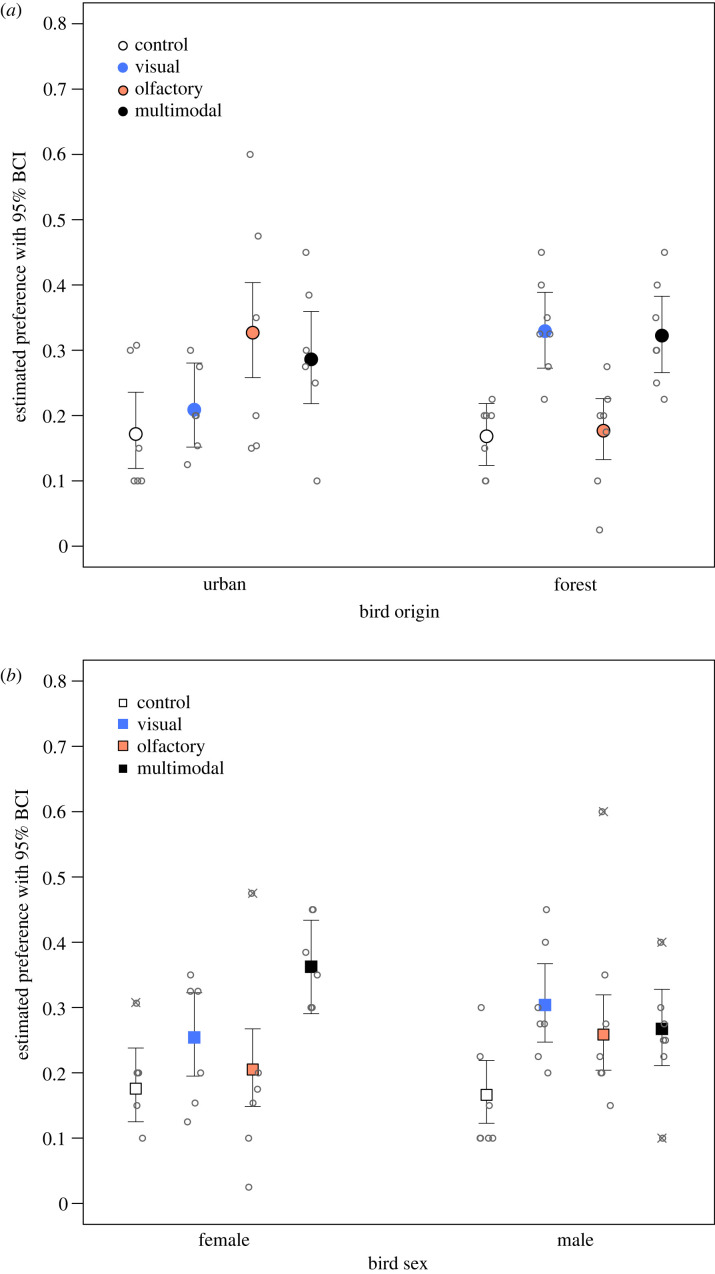
Model estimated preference (frequency of choices) of great tits for multimodal foraging cues and their individual components, with 95% BCIs. Preferences for birds of urban and forest origin (*a*) and females and males (*b*) are shown as proportion of visits to perches baited with multimodal (colour + odour), only visual (colour), only olfactory (odour), and no cues (control). Individual observations (*n* = 52) are shown as grey circles—for each of the 13 individuals, the proportion of visits to each type of cue is presented; potential outliers are marked with ×. All pairwise contrasts for which the probability of a difference exceeded 95% are shown in figure 2.

We found a clear difference in how urban and forest birds prioritized visual versus olfactory cues (figure 2 ‘origin’, ‘urban’, ‘forest’; figure 4*a*). Urban birds had similarly high levels of choice for the two cues that contained olfactory information (i.e. olfactory and multimodal), while forest birds preferred the cues with visual information (i.e. visual and multimodal). These choices were similarly high when compared to controls (figure 4*a*). The birds showed a considerable variation in preferences, particularly the urban birds, but no potential outliers were detected in this analysis.

#### 3.2.3. Sex differences

Female great tits had higher preference for multimodal cues than males, and female preference for multimodal cues was greater than for visual and olfactory cues alone (figures 2 and 4*b*). Males showed clear preferences to any type of foraging cues over controls, as indicated by higher proportion of choices for these treatments (figure 4*b*). The largest difference between the sexes was in their preference for the multimodal cues (females > males; figure 2 ‘sex’, ‘female’, ‘male’). We detected a couple of outliers (figure 4*b*), which may have influenced the estimated level of preference for olfactory cues; however, the outliers were similar for both sexes and thus did not influence the comparison between them.

The outcome of analyses was similar, irrespective of whether we used each cue separation test as an individual sample (*N* = 20) or each bird individual as a sample (*N* = 13). The estimated preference levels for the tested groups (‘urban’ and ‘forest’, ‘female’ and ‘male’) were nearly identical for both analyses and the assessment of ‘significance’ of the differences between the groups remained the same. The estimated preference values and confidence intervals for all analyses, including those for confounding factors, are presented in electronic supplementary material, appendix S4.

## 4. Discussion

Even though vision is regarded as the predominant sense in most birds [1], the great tits in our experiment responded to both visual and olfactory components of the multimodal foraging cues. We found limited evidence that the birds perceived the multimodal information in an integrated way, even though female great tits showed a higher preference for the multimodal cues. Instead, we found support for sensory prioritization and that modality preferences differed both between birds of urban and forest origin, and between sexes.

Multimodal information often facilitates behavioural responses like learning or reaction speed via multisensory integration; yet, this can be context-dependent and influenced by environmental noise [4], relative cue frequency (rats [5]), stimulus properties (bees [26]) or previous experience and expectations of the perceiver (humans [46], fruit flies [6]). In a learning experiment using the same experimental setup, but a larger number of individuals, we found no differences in how fast birds learned multimodal and unimodal cues, neither overall, between urban and forest birds, nor between sexes [28]. However, birds learned a second cue faster than a first one, particularly if the second cue was multimodal, perhaps suggesting that multimodal information helped them to generalize the task [28]. In the present experiment, we did not find an overall higher preference for multimodal cues, as for all birds combined, visual cues had similar preference. We also found little evidence that the multimodal cues were preferred initially during test trials, which indicates that great tits did not perceive the multimodal cues as more salient. Higher salience of multimodal cues would mean that they can efficiently attract attention in a bottom-up fashion, which is often observed as faster learning or higher preference in insects [8]. We found no indication for such effects, suggesting that the multimodal information did not facilitate great tit foraging behaviour. Thus, our results indicate that integration of visual and olfactory information via bottom-up (signal-driven) mechanisms as described for insects [3,8,24,25] might not occur in great tits.

When learning multimodal cues, the birds appear to have focused on either visual (forest birds) or olfactory (urban birds) cue components. However, female great tits showed a clear preference for multimodal over unimodal cues, and this needs to be investigated further to assess under which conditions multimodal information may specifically facilitate female foraging behaviour. More detailed experiments that manipulate the relative detectability of each modality, e.g. by adding environmental noise, are needed for a deeper understanding of how vision and olfaction interact in birds. Also, measuring the latency to locate food, or the amount of food obtained over a longer time period in a more complex experimental environment could test whether use of multimodal information can increase bird foraging efficiency.

In birds, an increasing number of studies indicate that olfaction sometimes can be as important as vision [15,16,29,47–49]. Yet, the two senses have rarely been simultaneously assessed [47,48,50]. We found differential prioritization of visual and olfactory foraging cues for birds of different sex and habitat of origin. The previously reported lack of differences in learning speed for these cues [28] indicates that visual and olfactory cues did not differ substantially in detectability to the birds and that both modalities were appropriate for the foraging task (‘modality appropriateness’ [2,7]), as birds passed the learning task with similar efficiency. Therefore, the variation in preferences we observed likely results from individuals voluntarily prioritizing the senses differently. This is an exciting finding, because the relative roles of vision and olfaction for foraging terrestrial birds are currently poorly understood. The use of visual and olfactory cues in foraging can be spatially scale-dependent in Procellariiform seabirds [49] or vary between specialized and opportunistically foraging species of scavenging raptors [48]. In detection and learning tasks, pigeons trained in an operant conditioning procedure selectively attended to vision over olfaction [51], while the ability of great tits to discriminate or learn olfactory and visual foraging cues appears to be similar [28,29]. Whether individuals of the same species can use vision and olfaction differently in different contexts has not been investigated previously. Our findings, therefore, contribute to a broader understanding of multimodal perception, which has been limited by the small number of taxa studied [4,8]. Assessing variation across populations and along urbanization gradient to explain what environmental factors drive these patterns presents a new challenge in the development of this field.

Contrary to our expectation, urban birds showed a preference for olfaction over vision when foraging within our experiment, while forest birds preferred visual cues. One potential explanation for why urban birds may benefit by prioritizing olfactory cues is improved ability to localize anthropogenic food sources. Urban environments are more heterogeneous than natural habitats at small spatial scales [52–55]. We may speculate that the visual heterogeneity of urban habitats is reduced in winter due to lack of green foliage, while the existing olfactory contrasts might be increased due to lack of volatile compounds released by plants. Thus, exploiting smell in particular could be adaptive for efficiently tracking anthropogenic food availability during winter. Whether anthropogenic food sources are important for species like the great tit may be questionable, even though some studies have implied a potential positive link between human activity and nestling condition [20]. To our knowledge, there are no studies that have tracked the movement patterns of individual birds in urban environments across seasons. Until such studies are performed, we will not fully understand what cues guide foraging behaviour of these birds at different times of the year.

Urban birds may be able to switch their attention more flexibly between visual and olfactory stimuli, since they usually are more exploratory and proactive compared to forest birds [56]. In that case, urban individuals may have prioritized olfactory cues because they found them to be more reliable based on previous experiences within their habitat. Attention is an important top-down driver of sensory prioritization linked to previous experiences of individuals, and this process shows high flexibility, compared to bottom-up processes that are more or less automatic [2]. We observed large variation in olfactory preferences of individual birds, which further suggests that great tits’ use of olfaction is flexible and there are probably additional factors influencing it, like personality, attention and previous experiences. Thus, studies on a larger number of birds are necessary to explore both cognitive and ecological processes that influence bird sensory preferences in urban and forest habitats and to assess potential habitat–sex interactions.

Our results did not support the expectation that urban air pollution may negatively influence olfactory preferences of urban birds. A possible explanation may be that the pollution levels in our studied urban area were not high enough to significantly influence bird ability to detect relevant habitat odours. Urbanization occurs along a gradient, and we may expect varying responses in bird traits in response to different levels of urbanization [20]. Another explanation may be that our study is a snapshot of preferences in time, and does not provide information on whether these preferences are stable or show seasonal changes between urban and forest birds. Thus, it is still possible that olfactory sensory pollution in urban settings may influence preferences at higher levels of pollution or at other times of the year. Olfactory function is linked to breeding activity in male European starlings (*Sturnus vulgaris*) [57] and testosterone level in male blue tits [22], suggesting probable seasonal variation in sensitivity. In addition, seasonal changes and sex differences in preen oil composition have been reported for many bird species, indicating increased importance of olfaction during breeding [58]. Therefore, attention towards olfactory stimuli in birds might increase at the start of breeding season during bud burst in spring, when plants start producing HIPVs [22]. If this is the case, then it is still possible that bird detection of HIPVs is hindered in urban habitats and their relative reliance on these specific olfactory cues may be lower. Whether air pollution could partly be responsible for reduced bird foraging efficiency on caterpillars in urban habitats, or whether this is only due to lower prey availability [18] needs to be addressed in future field experiments.

Female great tits prioritized the multimodal cues that they had previously learned, while males approached both the original cue and its individual components at a similar rate. Female preference for multimodal information could be linked to sexual selection if females attend to multiple male signals simultaneously [59]. However, multimodal male foraging displays in fowl (*Gallus gallus*) did not facilitate a stronger female response, as hens perceived visual and acoustic components of the multimodal display as redundant [27]. To our knowledge female perception of multimodal mating signals has not been empirically tested in great tits. For different components of colour signals, both female and male mate choice has been documented in the great tit [60,61], suggesting that both sexes may need to attend to multiple signals that indicate partner quality. It is possible that multisensory integration of visual and olfactory information increases the perceived cue salience during cue learning in females, but not in males. However, our previous experiment showed no differences in learning speed for either of the sexes [28]. The observed preference patterns may therefore suggest that females do not learn faster, but that they are more precise learners than males. Evidence indicates that observational learning performance is higher in female great tits, likely because females have lower rank at food sources and being more observant allows them to locate food hidden by hoarding species during winter [30]. In line with this, low ranked individuals of domestic fowl (*G. gallus domesticus*), particularly females, were better at a social cognitive task, which requires good observational skills [62]. Due to higher access to food, the motivation for learning and food retrieval might be lower in males than females [30], which conforms to our observation that males differentiated between cue and control, but foraged more randomly with respect of which type of cue was present. Thus, different preferences between the sexes may be due to different selection pressures on female and male learning and foraging strategies [30], which could lead to different levels of attention to the task and result in different prioritization patterns. These prioritization patterns call for the need to further explore top-down influence of attention and previous experiences on multisensory integration in this cognitively advanced bird species.

## 5. Conclusion

We found no support that multimodal information facilitated perception of foraging cues in great tits, at least via bottom-up signal-driven mechanisms of multisensory integration. Instead, we found that foraging preferences for visual and olfactory components of multimodal cues differed for urban and forest great tits, as well as for females and males, demonstrating that individual birds of the same species can prioritize sensory cues from different modalities in their foraging behaviour. Our study indicates that environmental context and sex are two potential drivers of sensory preferences in birds. Despite this, the cognitive and ecological mechanisms behind these patterns need to be investigated further, particularly top-down influence via selective attention shaped by previous experiences, as these have also been identified as important modulators of multisensory integration in other taxa. In addition, the implications of these preferences for bird foraging efficiency in the wild need to be tested in field experiments with more individuals and across populations. This study adds knowledge on multimodal perception and integration of olfactory and visual cues, which has so far only been studied in a limited number of taxa, and not previously assessed in birds. Our findings also identify potential urbanization effects on animal olfactory behaviour, which has largely been ignored by sensory pollution studies.

## Data Availability

The original data and code used in this paper are included in the electronic supplementary material [63].
